# Safety and feasibility of a novel recanalization technique using guidewire puncture under cholangioscopy for complete biliary stricture after liver transplantation

**DOI:** 10.1038/s41598-023-31475-1

**Published:** 2023-03-25

**Authors:** Wei Zhang, Hao Sun, Dinghui Dong, Yu Li

**Affiliations:** 1 Department of Hepatobiliary Surgery, The First Affiliated Hospital of Xi’an Jiaotong University, No. 277 Yanta West Road, Xi’an, 710061 Shaanxi Province China; 2 National Local Joint Engineering Research Center for Precision Surgery and Regenerative Medicine, Xi’an, 710061 China; 3 Shaanxi Province Center for Regenerative Medicine and Surgery Engineering Research, Xi’an, 710061 China

**Keywords:** Gastroenterology, Hepatology

## Abstract

Cholangioscopy is reportedly useful for selective guidewire placement across difficult biliary strictures, but few methods are available for complete stricture of biliary anastomosis. This study aimed to propose a guidewire puncture technique to recanalize totally obstructed anastomosis and discuss its safety and feasibility. From January 2015 to December 2021, a total of 11 patients with complete biliary anastomotic stricture after liver transplantation were enrolled. These patients underwent peroral single operator cholangioscopy (SpyGlass), whereas two failed cases on SpyGlass finally underwent percutaneous transhepatic cholangioscopy (PTCS). The steps of the recanalization technique were as follows: the stricture was viewed carefully to detect the closure point (CP) of the scar endoscopically, then the CP was targeted by the hard tip of the guidewire and broke through under guidance of the cholangioscope and fluoroscope. Complete occlusions were confirmed by SpyGlass in all cases. A total of 13 hard-tip guidewire punctures were performed under cholangioscopy, and ten punctures were successful (technical success rate, 76.9% [10/13]). After recanalization of the occluded anastomosis, plastic stent or metallic stent was deployed in three and seven patients, respectively. No procedure-related complications occurred during or after the cholangioscopy-assisted guidewire puncture. After a mean follow-up of 12 months, stents had been removed in five patients. The other six patients were still receiving stent treatment. This study demonstrated that the guidewire puncture technique under cholangioscopy is safe and feasible for complete stricture of biliary anastomosis, and the success rate is satisfactory.

## Introduction

Benign biliary anastomotic stricture, including bilio-biliary or bilio-enteric anastomotic stricture, is a common complication after biliary surgery, it is seen in patients after liver transplantation (LT) or Roux-en-Y cholangiojejunostomy. The causes are related to suture technique, bile leakage, local ischemia, infection, and other factors^1,2^. Endoscopic retrograde cholangiopancreatography (ERCP) or percutaneous transhepatic biliary drainage (PTCD) has largely replaced surgery as the first management of biliary anastomotic strictures, with a successful rate of 60–100%^3,4^. The key of ERCP or PTCD is traversing the stricture by a guidewire^5^, which allows for subsequent dilation or stenting.

As the “third” eye of hepatobiliary surgeons, the cholangioscope can not only observe intra-ductal lesions directly, but also complete therapeutic procedures, such as stone removal, biopsy, stricture expansion, etc. At present, clinicians can enter the biliary system through three ways: percutaneous transhepatic cholangioscopy (PTCS), percutaneous postoperative sinus cholangioscopy, and per-oral cholangioscopy. Numerous studies have reported that cholangioscopy successfully assisted guidewire placement for ERCP failed strictures^6–8^; however, few of them focused on complete strictures. Although needle-knife puncture^9^ and magnetic compression anastomosis^10^ have been proposed as nonsurgical alternatives, both require special accessories or more invasive procedures and have a high risk of complications. Cholangioscopy-assisted guidewire puncture technique for complete post-transplant bile duct stricture has not been reported previously. As a novel and simple alternative, we describe a puncture technique that only uses a guidewire for completely occluded anastomosis in this study and analyze its feasibility and safety.

## Patients and methods

### Ethics statements

This study was approved by the Medical Ethics Committee of the First Affiliated Hospital of Xi’an Jiaotong University (Xi’an, China). All procedures involving human participants were in accordance with the ethical standards of the institutional research committee and with the 1964 Helsinki Declaration and its later amendments. Written informed consent was obtained from all enrolled patients.

### Study design and patients

**Figure 1:**
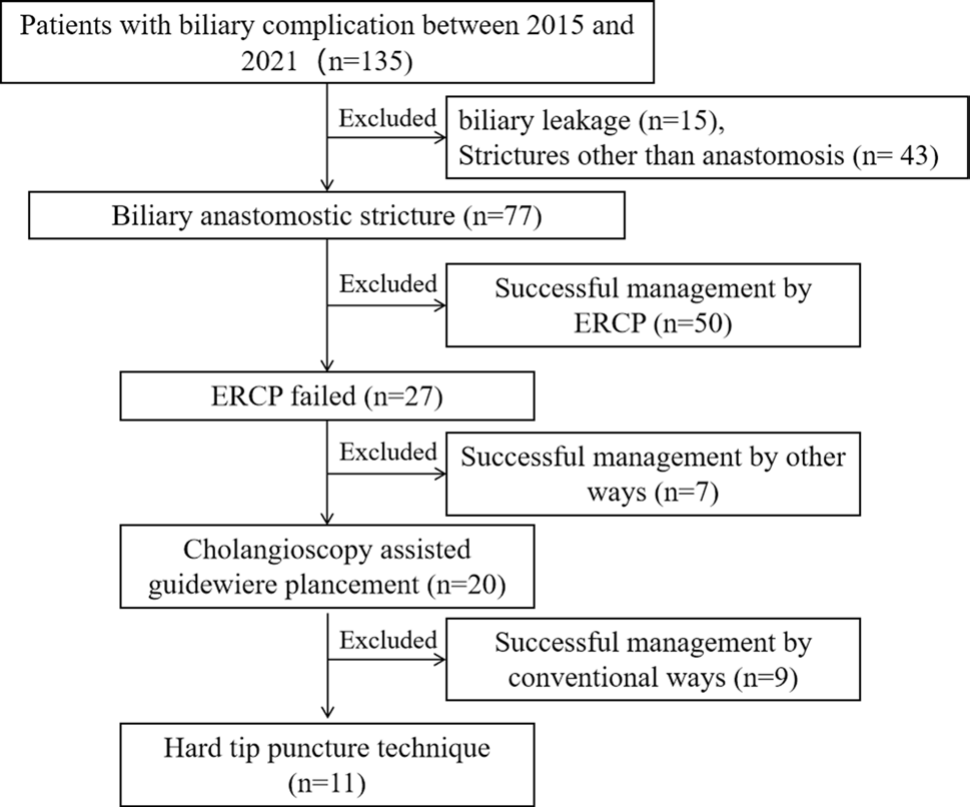
Flow diagram for enrollment of patients in the study. *ERCP* endoscopic retrograde cholangiopancreatography.

Between January 2015 and December 2021, a total of 870 patients underwent orthotopic liver transplantation (OLT) at the First Affiliated Hospital of Xi’an Jiaotong University. All grafts were donated after circulatory death or brain death. Bile duct reconstruction was established with duct-to-duct anastomosis without a T-tube (n = 780, 89.6%), with a T-tube (n = 80, 9.2%) or with an internal stent (n = 10, 1.2%). A total of 135 (15.5%) patients developed biliary complications after surgery. Inclusion criteria of the retrospective study were as follows: (1) biliary anastomotic stricture (AS); (2) conventional ERCP or PTCD failed; and (3) a complete stricture confirmed by cholangioscopy. Biliary leakage, non-anastomotic strictures (NAS), incomplete stricutres or strictures treated successfully by other ways were excluded, and a total of 11 patients were enrolled in this study (Fig. 1).

### Technical tips for performing the guidewire puncture technique under single operator cholangioscopy

Patients were under ERCP under intravenous anesthesia. A duodenoscope (TJF260; Olympus Optical, Tokyo, Japan) was used. After successful papillary cannulation, endoscopic sphincterotomy was performed. Then, the SpyGlass DS (Boston Scientific, Marlborough, MA, USA) or the eyeMax (Micro-Tech, Nanjing, JiangSu, China) was inserted into the bile duct over guidewire. Using normal saline injection, the biliary stricture was observed.

**Figure 2:**
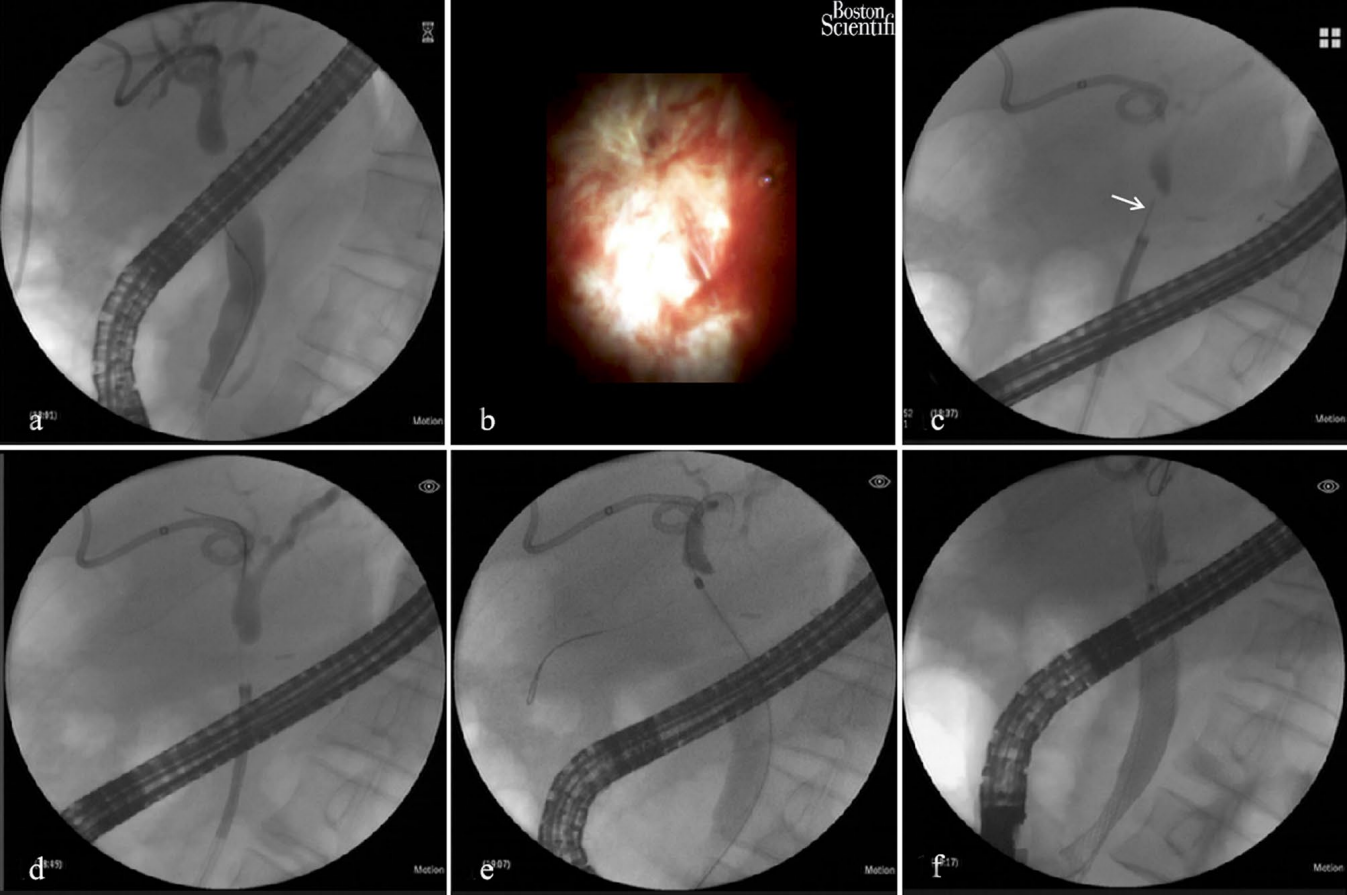
SpyGlass-assisted puncture of the complete biliary anastomotic strictures using the hard tip of the guidewire. (**a**) The stricture is impassable by conventional ERCP methods. The cholangiography, which is performed though PTCD and ERCP at the same time, implies two disconnected stumps. (**b**) The stricture is completely occlusive under SpyGlass. (**c**) Hard-tip (arrow) puncture of the stricture is performed under guidance of both SpyGlass and fluoroscopy. (**d**) Recanalization is successful, as indicated by the visible graft duct. (**e**) The stricture is expanded with 8 Fr dilating bougie. (**f**) A fully covered self-expanding metallic stent is deployed over the guidewire.

Once a complete stricture was confirmed by cholangioscopy, it was impossible to advance a guidewire through the AS conventionally; therefore, a puncture technique using hard tip of guidewire was proposed (Fig. 2). The stricture was viewed carefully to detect the closure point (CP) of the scar, which could be central or eccentric and usually present at the weakest site. The hard tip of a guidewire (Jagwire, 0.035 inch, Boston Scientific, USA) was advanced through the working channel to the top of the cholangioscope. Under the guidance of preoperative MRCP and real-time X-ray fluoroscopy, the cholangioscope was adjusted to the deemed axial direction of the opposite tract, and then the CP was punctured by the hard tip using reasonable force. If breakthrough was successful, a sudden decrease in resistance would be felt, and sometimes bile could be seen flowing through the puncture orifice (Supplementary Video S1). Cholangiography was performed to confirm recanalization through a 5-Fr bougies (Cook Medical, Bloomington, Indiana) advanced over the guidewire. The tract was dilated with cylinder balloon (CRE Wireguided, diameter 8, 9, 10 mm, work pressure 3, 5.5, 9 ATM, Boston Scientific, USA). Finally, one out of two stents: fully covered self-expandable metallic stent (WallFlex, diameter: 10 mm, length: 60 or 80 mm, Boston Scientific, USA) or plastic stent (Advanix-M00534280, diameter: 8.5-Fr, length 9 cm or 12 cm, Boston Scientific, USA) was placed across the stricture.

Complications related to cholangioscopy, guidewire puncture, and ERCP were recorded.

### Follow-up and data collection

The biliary stent was exchanged every 4 months and removed 12 or 6 months later. The removal of biliary stent required fulfillment of the following criteria^11–13^. First, there was no stricture demonstrated on the cholangiography, or there was still a minimal stricture, however, a 12 mm extraction balloon could easily pass through the anastomosis. Second, the mucosa around the anastomosis was smooth without scar and edema under endoscopic observation. Finally, patients had no related symptoms and abnormal biochemical examinations. Patients were followed by outpatient or telephone calls until June 2022. Clinical symptoms (jaundice, pruritus, abdominal pain, etc.), routine blood test results, liver and kidney function, and abdominal ultrasound findings were recorded.

### Statistical analysis

The SPSS 22.0 statistical software was used for analysis. Normally distributed quantitative data were represented using X ± S, and intergroup comparison was performed using the t-test. Non-normally distributed quantitative data were represented using M (range), and intergroup comparisons were made using the Mann–Whitney U test.

## Results

The demographic and clinical data of the enrolled 11 patients are summarized in Table 1. Of the patients, nine were men. Patients mean age was 53 ± 11.7 years. At presentation, all patients had jaundice, two had pain, and two had fever. The duration to presentation after surgery was 12 months (range, 8–30). Before receiving cholangioscopy, 11 patients underwent a total of 25 unsuccessful procedures, including 19 ERCPs and six PTCDs.

**Table 1:** The demographic and clinical data of the patients.

Case no	Sex/age	Duration from surgery to presentation (m)	Tbil (mg/dL)	Angle between superior and inferior duct on MRCP	Previous failed procedure	Central/eccentric scar	Puncture technique	Stent type	Stent-off (m)/recurrence
1	M/34	15	1.6	24.3°	ERCP × 1	Central	Success	FCSEMS	10/No
2	M/62	8	3.2	20°	ERCP × 1	Central	Success	FCSEMS	11/No
3	M/52	12	6.4	14°	ERCP × 1	Eccentric	Success	PS	5/NA
4	M/48	10	1.8	16°	ERCP × 2	Central	Success	PS	7/NA
5	M/60	8	6.4	11°	ERCP × 2	Eccentric	Success	FCSEMS	8/No
6	F/61	16	5.6	17°	ERCP × 3	Central	Success	FCSEMS	No/NA
					PTCD × 2				
7^a^	F/50	10	4.9	12°	ERCP × 2	Eccentric	Success	PS	No/NA
8^a^	M/69	30	9.4	24°	ERCP × 2	Eccentric	Success	FCSEMS	No/NA
9	M/32	8	2.6	18°	ERCP × 2	Central	Success	FCSEMS	No/NA
10	M/62	15	8.7	15°	PTCD × 1	Eccentric	Success	FCSEMS	No/NA
11^b^	M/55	18	5.2	35°	ERCP × 3	Eccentric	No	–	No/NA
					PTCD × 2				

*Tbil* total bilirubin, *ERCP* endoscopic retrograde cholangiopancreatography, *PTCD* percutaneous transhepatic cholangial drainage, *PS* plastic stent, *FCSEMS* fully covered self-expandable metallic stent, *NA* not available.

^a^managed with hard tip puncture technique under percutaneous transhepatic cholangioscope successfully.

^b^a fistula was found between the biliary anastomosis and the duodenum, across which a plastic stent was placed.

Total occlusions were confirmed by the single operator cholangioscopy in all cases, and five of those were central contractures. Primary punctures were performed by the hard-tip of guidewire under direct visualization with good orientation for all patients; however, in three cases (Case 7, 8 and 11), the punctures failed, which may have been caused by the unobvious CP or the scar thickness at the penetrated site. There were no episodes of extravasation of contrast material or guidewire outside the duct lumen.

**Figure 3:**
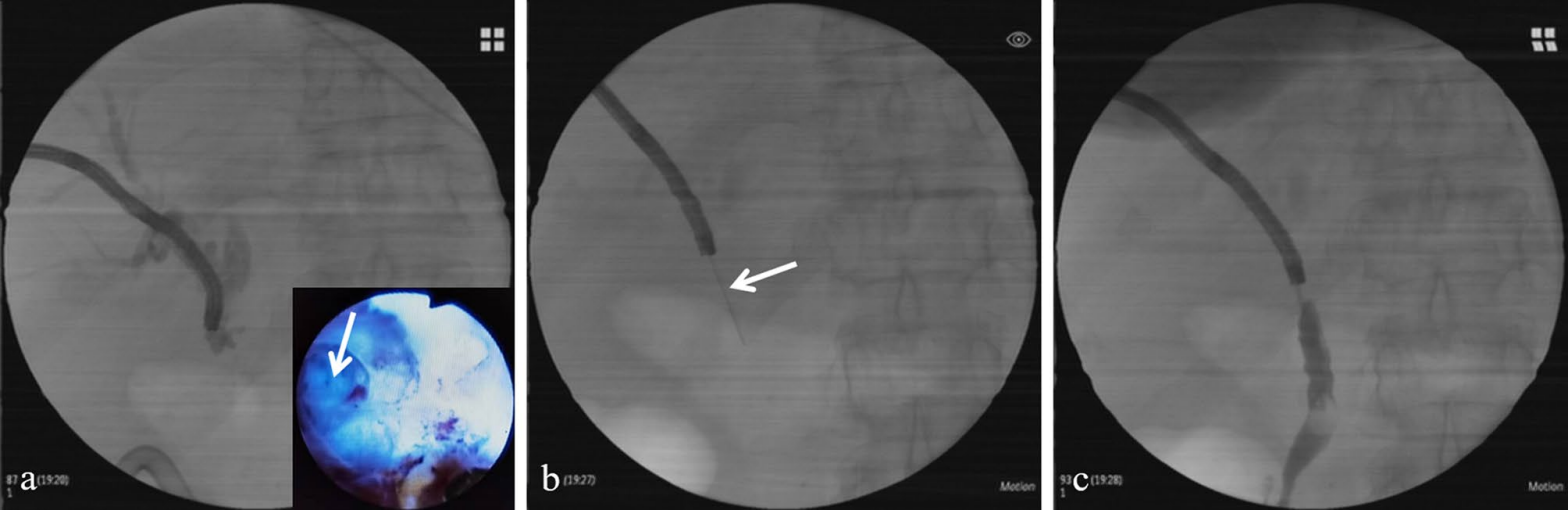
PTCS-assisted puncture of the complete biliary anastomotic strictures using the hard tip of the guidewire. (**a**) The bilio-biliary anastomosis is an eccentric occlusion (arrow) under PTCS; although contrast agent was injected, the distal duct does not become visible. (**b**) Hard-tip puncture (arrow) of the stricture is conducted under PTCS. (**c**) Recanalization is confirmed by the visible distal duct through radiography. *PTCS* percutaneous transhepatic cholangioscopy.

The three patients with failure underwent with the rendezvous technique combining PTCD and ERCP. Only one case completed stent placement, and a plastic stent was deployed across the fistula between the biliary anastomosis and the duodenum. The other two strictures could not be traversed though PTCD, and the hard tip puncture under percutaneous cholangioscopy succeeded for them (Fig. 3).

Overall, 11 patients received 13 guidewire punctures under cholangioscopy, and ten punctures were successful (technical success rate, 76.9% [10/13]). After recanalization of the occluded anastomosis, plastic stent, or metallic stent was deployed in three and seven patients, respectively.

There was mild cholangitis in two patient that resolved with antibiotic therapy. There were no immediate or delayed complications due to guidewire puncture, such as bleeding, perforation, and bile leakage.

After a follow-up of 12 months (range, 6–30 months), stents had been removed in five patients, who had no stricture recurrence at the end of follow-up. The other six patients were still receiving regular ERCP treatment.

## Discussion

This study proposed a simple recanalization technique for complete stricture of biliary anastomosis. The technique was performed in 11 indicated patients with a success rate of 75%, and no related complications were observed after the procedure. The results suggested that guidewire puncture using a hard tip under cholangioscopy is a safe, feasible method for those kinds of patients.

The primary technical success rate of ERCP for benign biliary stricture is over 90%^11,14^. Inability of the guidewire to pass through the stricture was the main reason for failure^5^. Traditionally, different guidewires, occlusion balloons, or rotatable sphincterotome were alternative methods to improve the possibility of traversing^15^. Nevertheless, these techniques mentioned previously were performed blindly under X-ray fluoroscopy, which challenged the experience of the endoscopists to judge the possible small opening of the stricture.

The SpyGlass system could provide direct visualization of the orifice of the stricture and avoid the need for a repeat endoscopic/percutaneous approach or revision surgery. However, the stricture type, degree of stenosis, and angulation or crane neck deformity of the bile duct would influence the placement of the guidewire even under the SpyGlass system. Bokemeyer et al.^6^ reported 30 failed cases of ERCP, and only 70% of those were traversed under the Spyglass system, and the success rate of benign strictures was significantly higher than that of malignant ones (86.2% versus 44.2%). Woo et al.^8^ could view 93.3% of anastomotic strictures after living donor LT under the Spyglass system. Because of the sharp angulation between the proximal and distal ducts, the passage of the guidewire was only achieved in 60% of patients. In the case of complete stricture, it was impossible for only the guidewire to traverse a biliary stricture without any other accessories. In this study, the hard tip of the guidewire was used to manage this difficulty, which was not reported before. Unlike the soft tip, the hard one was strong enough to maintain its straightness while breaking through the fibrotic tissue, and eight cases were successful without any complication. The key to this technique was puncturing the CP toward the deemed direction of the proximal bile duct. Another needle puncture technique under ERCP guidance has been reported^16^; it was performed blindly and required straight alignment of the bile duct above and below the stricture. In this study, the puncture was visualized, and the direction could be adjusted by cholangioscope. In addition, it was difficult for the blunt hard tip to penetrate the duct wall or thick scar tissues, which ensured safety of the puncture. However, accurate judgement of the CP and thickness of the stricture were key factors related to success, as demonstrated by the failed cases in this study.

PTCD was a commonly used method for bilio-enteric anastomotic stricture or failed ERCP cases^17,18^. The maneuver of advancing the guidewire through the stricture percutaneously was challenging, with a reported success rate of 59.3%^19^. PTCS could be performed to enter the intrahepatic bile duct to observe lesions and assist guidewire placement for difficult strictures^20,21^. Nevertheless, the goal of total obstruction was still impossible for PTCS. A “sandwich” technique has been reported^22,23^: the occluded anastomosis is approached from two sides by simultaneous PTCS and peroral enteroscopy. Toshio Fujisawa et al.^22^ used the soft tip of the guidewire to puncture the scar tissue under enteroscopic guidance. Steinbrück et al.^23^ cut through the separating scar tissue layer with a needle-knife under endoscopic and fluoroscopic control using diaphanoscopy, which was successful in three patients. Although enteroscopy was useful to monitor the puncture or mark the target, it was difficult and time-consuming to reach the site of cholangiojejunostomy^24^, so the “sandwich” technique may not be necessary. For example, Lim et al. created an artificial bilioenteric fistula using a needle-knife papillotome only during PTCS^25^. Indeed, the risk of iatrogenic perforation should be acknowledged while using the needle-knife. Interestingly, two cases failed under the SpyGlass system but were successfully recanalized under PTCS with hard tip of the guidewire puncture; this may have been caused by the fact that the CP of the stricture was more recognizable on the superior side than on the inferior side. These cases indicated that our proposed puncture technique can also potentially be utilized via PTCS for occluded bilio-enteric anastomoses or faild cases of ERCP with SpyGlass.

Compared with the sharp puncture tools, such as needle-knife (visually or blindly), the hard-tip guidewire puncture under cholangioscopy is safer. The most serious complication of needle-knife puncture or electrotomy is hemobilia, with a reported incidence of 6.0%^16,25^ and excessive electrotomy could even cause hepatic abscess and bile leakage. However, in this study, no complications related to puncture procedure, e.g., biliary bleeding, bile leakage, perforation, or vascular injury, were observed. The small caliber of the guidewire, blunt tip, and cholangioscopy assistance may ensure safety.

There were some limitations of this technique. (1) The optimal thickness of the stricture was hard to determine. If the stricture was too severe, a needle puncture or magnetic compression anastomosis may be an alternative to this technique. (2) There was a shortage of corresponding accessories. The hard tip of the guidewire was too hard to be passed through the working channel while the body of cholangioscope was curved enough. Thus, a compatible catheter or needle should be developed. (3) This study was a case series; therefore, more cases and experiences at other centers are needed to verify the method in terms of short- and long-term results.

This study shows that the cholangioscopy-assisted guidewire puncture technique is a safe and feasible method for managing complete stricture of biliary anastomosis. Larger multicenter case series are needed to further study the long-term safety and outcomes of this technique.

## Supplementary Information

Supplementary Legends.

Supplementary Video S1.

## Data Availability

The datasets used and analysed during the current study are available from the corresponding author on reasonable request.
