# Supplementary material for: Safety and feasibility of a novel recanalization technique using guidewire puncture under cholangioscopy for complete biliary stricture after liver transplantation
Source: Sci Rep. 2023 Mar 25;13:4874. doi: 10.1038/s41598-023-31475-1 (PMC10039916; doi:10.1038/s41598-023-31475-1)
Supplement: Supplementary file 1 — Supplementary Legends. [file 41598_2023_31475_MOESM1_ESM.docx]

**Video legend**

Video S1. Novel guidewire puncture technique under SpyGlass for a complicated biliary anastomotic stricture following liver transplantation.
